# Surgery for Cystic Pancreatic Lesions in the Post-Sendai Era: A Single Institution Experience

**DOI:** 10.1155/2015/847837

**Published:** 2015-03-19

**Authors:** Jörg Kleeff, Christoph Michalski, Bo Kong, Mert Erkan, Susanne Roth, Jens Siveke, Helmut Friess, Irene Esposito

**Affiliations:** ^1^Department of Surgery, Technische Universität München, Ismaninger Strasse 22, 81675 Munich, Germany; ^2^Department of Surgery, University of Heidelberg, Im Neuenheimer Feld 110, 69120 Heidelberg, Germany; ^3^Department of Surgery, Koc University School of Medicine, Rumelifeneri Yolu, Sariyer, 34450 Istanbul, Turkey; ^4^Department of Internal Medicine, Technische Universität München, Ismaninger Strasse 22, 81675 Munich, Germany; ^5^Institute of Pathology, Technische Universität München, Ismaninger Strasse 22, 81675 Munich, Germany; ^6^Institute of Pathology, Medical University of Innsbruck, Muellerstrasse 44, 6020 Innsbruck, Austria

## Abstract

*Introduction*. The management of cystic pancreatic lesions has changed in recent years as a result of increasing knowledge of their biological behaviour, better diagnostic options, and international guidelines. *Methods*. Retrospective analysis of a cohort of 86 patients operated for cystic pancreatic lesions during a seven-year period (2007–2014). *Results*. Final histopathology revealed 53 intraductal papillary mucinous neoplasms (19 branch duct IPMNs, 15 mixed type IPMNs, and 19 main duct IPMNs), 14 serous and 13 mucinous cystic neoplasms, 3 solid pseudopapillary neoplasms, and 3 other lesions. 4 cases displayed high grade intraepithelial neoplasia and 2 cases displayed invasive cancer. A pylorus-preserving partial duodenopancreatectomy was carried out in 27 patients, a total pancreatectomy was carried out in 9 patients, a left resection was carried out in 42 patients, and segmental resections and enucleations were carried out in 4 patients each. Overall postoperative morbidity and mortality were 40% and 2.3%, respectively. The preoperative diagnosis of a specific cystic tumor was accurate in 79% of patients and 9 patients (10%) could have avoided surgery with the correct preoperative diagnosis. *Conclusion*. Cystic pancreatic lesions are still a diagnostic challenge, requiring a dedicated multidisciplinary approach. The rate of malignancy is relatively small, whereas postoperative morbidity is substantial, underscoring the importance of adequate patient selection considering both the risk of surgery and the long term risk of malignancy.

## 1. Introduction

Pancreatic cystic lesions are a diagnostic challenge, particularly because they are increasingly often discovered on cross-sectional imaging [1, 2]. Cystic tumors of the pancreas may be precursors of pancreatic cancer, which is still one of the most difficult cancers to treat and has an extremely poor prognosis [3].

Most cystic lesions of the pancreas are neoplastic lesions and, of the far less frequent nonneoplastic cystic lesions, pseudocysts are the most common. Pseudocysts usually develop following an episode of acute pancreatitis or abdominal trauma, rarely are a diagnostic challenge, and can, in the case of symptoms or secondary problems, be treated by interventional and/or surgical means [4].

In contrast to pseudocysts, a precise diagnosis of neoplastic cystic lesions is often difficult. This is clinically relevant because treatment strategies differ widely depending on the underlying pathology [5]. There is general consensus that symptoms are an indication for intervention/surgery. However, incidental and not symptomatic cysts are more common and more challenging. Briefly, serous cystic neoplasms (SCN) are almost always benign and can be observed in most cases. In contrast, serous pseudopapillary neoplasms (SPN) have a relevant malignant potential and should be resected. Main duct intraductal papillary mucinous neoplasm (MD-IPMN) or mixed type IPMN also has a relevant malignant potential and should be resected, whereas branch duct (BD) IPMN has a malignant potential but the risk for progression to overt malignancy is not readily apparent [6]. A number of features have been identified that better stratify BD-IPMNs [5, 7]; yet the controversy regarding the best treatment strategy for this entity continues [5, 8–10].

Better diagnostic using high resolution cross-sectional imaging (CT, MRT) and endoscopic ultrasound/FNA as well as international guidelines have changed the approach to cystic pancreatic lesions in the last decade. Therefore, we retrospectively analysed resected cystic lesions of the pancreas in a tertiary referral center with special emphasis on the spectrum of lesions, the diagnostic accuracy, and the outcome.

## 2. Patients and Methods

The Institutional Review Board (IRB) approved prospective data collection (IRB#60/15). Analysis was conducted on an anonymized data set.

### 2.1. Database and Recorded Parameters

A prospective database for patients with pancreatic diseases has been established at our institution since 07/2007. The following parameters were recorded: diagnostics (EUS, CT, and MRT), cyst fluid analysis (including CEA and CA 19-9), patient history (including pain, weight loss, diabetes mellitus, and history of pancreatitis), preoperative blood parameters (including creatinine, ALAT, ASAT, bilirubin, WBC, platelets, CA1 9-9, and CEA), ASA score, operation details, histopathological details, postoperative complications according to the Clavien-Dindo classification [11], postoperative pancreatic fistula and bleeding according to the ISGP definition [12], and follow-up data.

### 2.2. Statistics

Continuous variables are reported as means ± SD or median (95% CI) and were compared using Student's *t*-test or Mann-Whitney *U* test, as appropriate. Categorical variables are summarized as frequency counts and percentages and were compared using Fischer's exact test or Pearson's chi-square test, as appropriate. A two-sided *P* value of <0.05 was considered as significant. All statistical analyses were performed using IBM SPSS v20 for Windows (IBM Inc., USA).

## 3. Results

From 07/2007 until 07/2014, 788 pancreatic resections were carried out at the Department of Surgery of the Technical University Munich, Germany. Of those, 86 resections were performed for cystic pancreatic lesions (11%). Cystic lesions that were not the indication for surgery but incidental findings at final histopathology (e.g., small side-branch IPMN in a case of advanced PDAC) were not included in this analysis.

There were 50 female and 36 male patients with a median age of 68 years (range: 13–86 years; Table 1). Cystic lesions were localized in all parts of the pancreas without any predominant localization (Table 1). The median size of the cyst at diagnosis was 3.3 cm (range 0.6–18 cm). 47/86 (55%) patients presented with symptoms: abdominal pain in 32 patients, weight loss in 19 patients, and jaundice in 3 patients (Table 1). Most patients were referred to our department with basic diagnostic procedures including abdominal ultrasound and/or CT/MRI scan. Our standard work-up included high resolution MD-CT or MRI scanning and in case of diagnostic uncertainty EUS with FNA for cytology and cyst fluid analysis. CT and/or MRI was carried out in 65/86 and 51/86 patients (Table 2). EUS was performed in 54/86 patients with FNA in 21 of those patients. Before 2012, indications for surgery for patients with cystic pancreatic lesions were decided primarily by the surgeons, on occasion together with the radiologists or gastroenterologists; however, no structured work-up was established for those patients. Preoperative diagnosis relied mainly on cross-sectional imaging; EUS was carried out infrequently. Since 2012 all patients with suspected pancreatic pathology were discussed in an interdisciplinary board and diagnostic and therapeutic strategies were documented. Diagnostic work-up included EUS with FNA for cytology and cyst fluid analysis for the majority of cases.

Preoperative diagnosis was main duct (MD) IPMN in 22 patients and mixed type IPMN in 7 patients. 17 patients were preoperatively diagnosed with branch duct (BD) IPMN, of whom 14 were Sendai [7] positive (size >3 cm in 7 patients, dilation of the main pancreatic duct in 4 patients, mural nodules and positive cytology in 1 patient each, and abdominal symptoms in 8 patients). For the remaining three patients, indication was resection of a neuroendocrine tumor of the Papilla of Vateri and cholecystectomy with concomitant resection of the cystic tumor in both and patient preference in one case. Preoperative diagnosis was MCN in 10 patients, SCN in 4 patients (all 4 patients complained about abdominal pain), and SPN in 3 patients. Indication for surgery were cystic lesion with suspicion of malignancy in 9 patients (2 patients with weight-loss and 1 patient with jaundice) and not further specified cystic lesions in 14 patients (8 with abdominal pain, 1 with weight loss, 3 with cystic lesions larger than 6 cm, and 2 patients' preference). Thus, applying current guidelines, there was an indication for resection in 78/86 (91%) patients. All patients without recommended indication were treated in the first period (until 2011). Thus in this period there was an indication for resection in 52/60 (87%) patients, compared to 26/26 patients in the second period (*P* = 0.048).

Regarding the size of the lesion, there were 18 cases of cystic lesions <2 cm. 12 of those were suspected BD-IPMN (see above) or mixed type IPMN. In 3 cases, there was suspicion of malignancy, 1 case was symptomatic, and 1 case each was diagnosed preoperatively as MCN and SPN, respectively.

As depicted in Table 2, a pylorus-preserving partial duodenopancreatectomy was carried out in 27 patients, a total pancreatectomy in 9 patients, a left resection in 42 patients, a segmental resection in 4 patients, and an enucleation in 4 patients. Median operating time was 260 min (range: 75–496 min). Overall postoperative morbidity was 40% with grade B/C pancreatic fistula of 11.6%. Mortality was 2.3% (2/86). The history of those two patients was as follows. The first patient was an 82-year-old male patient with suspected BD-IPMN with mural nodules. Final histology revealed BD-IPMN, gastric type, low grade IEN. The patient underwent a distal pancreatectomy, developed a grade C pancreatic fistula, and was revised several times for segmental bowel ischemia with subsequent peritonitis. He ultimately died of multiorgan failure. The second patient was a 77-year-old male patient with suspected mucinous neoplasia (EUS and cyst fluid analysis with CEA of 9638 ng/mL and CA 19-9 of 536 U/mL). Final histology revealed a ductal retention cyst and no neoplastic epithelium. The patient underwent a pylorus-preserving duodenopancreatectomy and developed a grade C bleeding with mass transfusion due to an aneurysm of the hepatic artery. He was immediately revised and the bleeding was controlled, but he died within 24 hours of the revision of multiorgan failure.

Postoperative histology is depicted in Table 3. There were 2 nonneoplastic lesions (one pseudocyst and one retention cyst). There were 14 serous and 13 mucinous cystic neoplasms, 3 solid pseudopapillary neoplasms, and one IPNB. The most common lesions were IPMNs (*n* = 53) with 19 BD-IPMNs, 15 mixed type IPMNs, and 19 MD-IPMNs. 4 cases displayed high grade intraepithelial neoplasia and 2 cases invasive (T1) cancer (Table 3). Altogether, there were 14 cases of SCN in this series. Only two cases were resected in the second time period: one for abdominal symptoms and one misdiagnosed as MCN despite preoperative EUS. In contrast, during the first time period, only 3 of the 12 cases were symptomatic and 9 cases were misdiagnosed (only 2 of them had preoperative EUS).

Analyzing pre- and postoperative diagnoses, there was a relevant diagnostic inaccuracy. Thus, of 22 patients with suspected MD-IPMN, 20 were confirmed, whereas the remaining were 1 BD-IPMN and 1 SCN. Similarly, of the 17 and 7 patients with suspected BD-IPMN and mixed type IPMN, 14 and 6 were confirmed, respectively. Two of the suspected BD-IPMN were MD-IPMN, and a third one was a mixed-type IPMN; 1 suspected mixed-type IPMN was a BD-IPMN. All patients operated for symptomatic suspected SCN (*n* = 4) were confirmed. Of the 10 patients operated for suspected MCN, 6 were confirmed, the remaining being 2 BD-IPMN, 1 SCN, and 1 retention cyst. Thus the suspected diagnosis of a specific cystic tumor was accurate in 79% (50/63) of patients. Comparing the two study periods, diagnostic accuracy increased slightly from 77% (30/39) to 83% (20/24; *P* = 0.2). In the 9 patients with cystic lesion and suspected malignancy, there were 2 cases of MD-IPMN (2 with high grade IEN and one T1 cancer), 1 BD-IPMN without high grade IEN, 2 SPN, 2 SCN, and 1 IPNB.

## 4. Discussion

In this study, we present a series of 86 resected cystic lesions of the pancreas out of 788 pancreatic resections that were carried out during the study period.

Cystic lesions of the pancreas are diagnosed with increasing frequency [1, 2] and are still a diagnostic challenge. The first question that has to be addressed is whether the cyst is neoplastic or not. Pseudocysts most often develop following an attack of acute pancreatitis or blunt abdominal trauma [13, 14] and the diagnosis is usually straightforward. In our series, there was only one pseudocyst that was a suspected neoplastic cyst preoperatively (1/86), supporting the aforementioned notion. In addition, there was one pancreatic retention cyst that was preoperatively mistaken for a neoplastic cyst. This is in line with data showing that nonneoplastic epithelial pancreatic cysts are rare and difficult to distinguish from cystic neoplasms [15].

Since 2012, we have established an interdisciplinary standardized preoperative work-up and documentation for cystic pancreatic lesion, including MRI/CT scan as well as EUS in all cases [16, 17], and EUS with FNA (cytology and cyst fluid analysis [5, 18, 19]) for cases of diagnostic uncertainty. Before that time, symptomatic patients with a cystic lesion were resected mostly without further diagnostic (i.e., EUS/FNA) work-up. As per current guidelines, there was a clear indication for resection in 78/86 patients (91%). Reasons for resection in the remaining patients included patient preference in 3 cases and cyst size (larger than 6 cm) in additional 3 cases. In 2 patients, the cysts were discovered during preoperative work-up for another indication and then resected during surgery.

In our study, a specific diagnosis was established preoperatively in 63 cases. Comparing preoperative diagnosis with the final postoperative histopathological report, there were 16 inaccurate diagnoses (25%). This is in line with data from centers with broad experience in cystic pancreatic lesions, where the overall inaccuracy rate was 22% in 476 patients [20], underscoring the fact that accurate diagnoses is still a challenge also in centers of excellence. In our series, taking also symptomatic lesion as an indication for surgery, 9 patients (10%) could potentially have avoided surgery with the correct preoperative diagnosis (1 pseudocyst, 1 retention cyst, 4 SCN, and 3 Sendai negative BD-IPMN).

Better preoperative diagnostic can be achieved by standardized interdisciplinary work-up, using MRT/CT as well as EUS, FNA with cytology, and cyst fluid analysis (CEA, CA19-9). Some studies have shown accuracy rates for EUS as high as 90% while others have shown that EUS is not sufficiently accurate to differentiate between benign and malignant lesions in most cases [16, 17]. Similarly, cyst fluid analysis (CEA, CA19-9) has been shown to differentiate with high specificity mucinous versus serous lesions and pseudocysts [19]. Interestingly, a combination of EUS, cytology, and cyst fluid analysis (CEA) is not better in differentiating between a mucinous and nonmucinous cyst than CEA alone [18], and cytology has its limitations as well [21]. Newer diagnostics such as KRAS or GNAS mutation analysis have not been validated in larger cohorts [22].

There is an ongoing debate regarding the risk of malignancy and thus the best approach to BD-IPMN, where, in contrast to MD-IPMN and mixed type IPMN, resection is tailored to specific criteria (Sendai and revised Sendai criteria [5, 7]). In our series, all BD-IPMN (17/19 Sendai positive) were benign, underscoring the findings of a systematic review demonstrating that malignancy is rare in Sendai positive BD-IPMN and virtually absent in Sendai negative BD-IPMN [9]. In addition, the preoperative distinction between different types of IPMN is difficult. Thus, in a recent series, 21% of patients with BD-IPMN had main duct involvement and 29% of those with suspected main duct involvement had none [23].

In our cohort, only 8 of the 66 patients with mucinous lesions (MCN and IPMN) had already high grade dysplasia or invasive cancer. The risk of low grade lesions to progress to malignancy is not known; yet data from other organs such as Barrett's esophagus show that most of these lesions do not progress to malignancy [24]. Cleary, if these lesions could be removed without risk, surgery would be therapy of choice in all cases. However, morbidity remains substantial and mortality is not nil in our and other series [25, 26]. Therefore, we need better methods to risk-stratify our patients, taking into account age and comorbidities, the extent of any potential resection, and the biology of the underlying tumor.

In conclusion, cystic pancreatic lesions still are a diagnostic challenge, requiring a dedicated multidisciplinary approach. The rate of malignancy is relatively small, whereas postoperative morbidity is not negligible. However, the number of premalignant lesions even in cases without preoperative suspicion is relatively high. Thus, adequate patient selection remains important considering both the risk of upfront surgery and the long term risk of malignancy. This is particularly true in younger patients where there may be a high cumulative risk of malignancy during lifetime.

## Figures and Tables

**Table 1:** Included patients, diagnostics, and symptoms (*n* = 86).

Gender	
Female	50
Male	36
Age (years)	
Median	68
Range	13–86
Localization	
Pancreatic head	30
Uncinate process	2
Pancreatic head/body	6
Pancreatic body	11
Pancreatic body/tail	3
Pancreatic tail	31
Whole pancreas	3
Imaging/diagnostics	
CT scan	65
MRI scan	51
EUS	54
Cyst fluid analysis	21
Symptoms	
Jaundice	3
Weight loss	19
Abdominal pain	32
h/o acute pancreatitis	18
Diabetes mellitus	18

**Table 2:** Operative and perioperative details (*n* = 86).

Type of operation	
pp Whipple	27
Total pancreatectomy	9
Segmental resection	4
Left resection	42
Enucleation	4
Operation time (min)	
Median	260
Range	75–496
Morbidity (Clavien-Dindo)	
1	5
2	12
3	13
4	2
5	2
Pancreatic fistula (ISGP)	
A	nd
B	6
C	4

nd: not determined.

**Table 3:** Histopathological characteristics (*n* = 86).

Pseudocyst	1
Retention cyst	1
Serous cystic neoplasia	14
Microcystic	8
Oligocystic	3
Macrocystic	1
ns	2
Solid pseudopapillary neoplasia	3
Mucinous cystic neoplasia	13
Low grade IEN	11
High grade IEN	2
IPMN	53
Side branch	
Low grade IEN	19
High grade IEN	0
Mixed type	
Low grade IEN	14
High grade IEN	0
Invasive cancer	1 with pT1 pN0
Main duct	
Low grade IEN	14
High grade IEN	4
Invasive cancer	1 with pT1 pN0
Gastric	27
Intestinal	17
Pancreatobiliary	1
Oncocytic	1
ns	7
IPNB	1

IPMN: intraductal papillarymucinous neoplasm; IPNB: intraductal papillary neoplasm of bile duct; IEN: intraepithelial neoplasia; ns: not specified.
